# Evaluation of miRNA-210 as a Prognostic Biomarker for Pre-eclampsia: A Case-Control Study

**DOI:** 10.7759/cureus.83453

**Published:** 2025-05-04

**Authors:** Kota Sai Meghana, Rajasri G Yaliwal, Gurushantappa S Kadakol, Shailaja R Bidri, Shreedevi Kori

**Affiliations:** 1 Department of Obstetrics and Gynecology, Shri BM Patil Medical College Hospital and Research Centre, Bijapur Lingayat District Educational Association (BLDE) (Deemed to be University), Vijayapura, IND; 2 Department of Genetics, Shri BM Patil Medical College Hospital and Research Centre, Bijapur Lingayat District Educational Association (BLDE) (Deemed to be University), Vijayapura, IND

**Keywords:** circulating mirnas, early prediction of pre-eclampsia, gene regulation in pregnancy, hypoxia-inducible factor (hif-1α), microrna expression, mirna-210, non-invasive diagnostic marker, placental ischemia, pre-eclampsia, rt pcr

## Abstract

Background: Pre-eclampsia (PE) is a complex, multisystem disorder occurring during pregnancy and remains a significant cause of maternal and perinatal morbidity and mortality. Despite its clinical importance, the underlying molecular mechanisms contributing to the development of PE are not fully understood. Specific microRNAs, such as miRNA-210, may play a crucial role in the pathophysiology of PE.

Objective: This study investigates the potential utility of miRNA-210 as a biomarker for the early detection and risk prediction of PE.

Materials and methods: This case-control study was conducted from April 2023 to February 2025 and involved PE and normotensive pregnant women admitted to the Department of Obstetrics and Gynecology at Shri BM Patil Medical College Hospital and Research Centre, Bijapur Lingayat District Educational Association (BLDE) (Deemed to be University), Vijayapura. Women who met the inclusion criteria were enrolled after providing written informed consent. A total of 102 pregnant women participated, divided into two groups: Group 1 included 51 women diagnosed with PE, and Group 2 comprised 51 normotensive pregnant women matched for age (±5 years), gestational age (±1 week), and obstetric score, serving as the control group. All participants underwent detailed history-taking, clinical examinations, and laboratory investigations, including coagulation profile, liver function test, renal function test, complete blood count, proteinuria evaluation, and miRNA-210 gene expression analysis via reverse transcription-polymerase chain reaction. A peripheral venous blood sample was collected at admission and submitted for genetic analysis. After genetic analysis, study parameters were compared, and maternal and neonatal outcomes were observed.

Results: Individuals with PE exhibited a statistically significant elevation in serum miRNA-210 levels (p<0.001) compared to the control group. miRNA-210 demonstrated a highly significant positive correlation with systolic blood pressure, mean arterial blood pressure, diastolic blood pressure, serum protein, serum uric acid, serum albumin, and serum alkaline phosphatase. However, no significant correlation was observed with hemoglobin percentage, platelet count, international normalized ratio, or serum creatinine. The area under the curve was 0.994, with a 95% confidence interval ranging from 0.985 to 1.0. At a cut-off value of 9.63-fold change, serum miRNA-210 levels showed a sensitivity of 100% and a specificity of 96.1%, with positive and negative predictive values of 96.2% and 100%, respectively.

Conclusions: Elevated serum miRNA-210 levels were observed in women with PE compared to normotensive pregnant women. Thus, serum miRNA-210 may serve as a non-invasive diagnostic and predictive biomarker in PE and contribute to a better understanding of its etiology.

## Introduction

Pre-eclampsia (PE) is one of the most serious pregnancy complications, affecting 2% to 8% of pregnant women. It is characterized by proteinuria and hypertension occurring after 20 weeks of gestation in previously normotensive women. PE poses a significant risk to both maternal and fetal health, particularly when it develops early in pregnancy [1,2]. Each year, approximately 76,000 women and 500,000 children die due to this condition [3]. Moreover, women in low-resource nations are at a higher risk of developing PE than those in high-resource nations.

The exact pathogenesis of PE remains incompletely understood. The prevailing hypothesis describes a biphasic mechanism: an initial abnormal trophoblast invasion of the maternal spiral arteries in early pregnancy leads to inadequate placental perfusion and persistent hypoxia. This, in turn, triggers a systemic maternal inflammatory and endothelial response in the second phase. MicroRNA-210 (miR-210), a hypoxia-induced miRNA, is upregulated in PE and contributes to the hypoxic response by impairing mitochondrial function, inhibiting trophoblast invasion, and promoting oxidative stress, further exacerbating placental dysfunction [3,4].

In the first phase, restricted trophoblastic infiltration leads to insufficient remodeling of the spiral arteries, causing chronic placental ischemia and hypoxia [4]. This then transitions into the second phase, characterized by the maternal response to pathological endothelial dysfunction and an imbalance of pro-angiogenic and anti-angiogenic factors, ultimately resulting in the clinical symptoms of the disease.

Endogenous miRNAs are small, noncoding RNAs, ranging from 20 to 24 nucleotides in length, that suppress the expression of specific genes post-transcriptionally. Pineles et al. reported novel findings on differential miRNA expression in the placentas of patients with PE, marking the first such report in the literature [5].

The overexpression of miRNA-210 allows hypoxia-inducible factors to modulate the cellular response to hypoxia by altering mitochondrial function, thereby promoting a shift from mitochondrial respiration to glycolysis. This process encompasses erythropoiesis, differentiation, angiogenesis, apoptosis, inflammation, cell proliferation, and metabolism [6].

Studies have shown that hypoxic conditions develop in PE, producing miRNA-210 via hypoxia-inducible factor-1α, thus establishing a link between PE and miRNA-210 [7]. Research also indicates that miRNA-210 may be a potential diagnostic and detection marker for early-stage PE [8]. Additionally, miRNA-210 is detectable in circulating blood, making plasma-based evaluation more feasible [9].

To explore the diagnostic utility of miRNA-210 in PE, we conducted a case-control study measuring its expression levels in maternal serum.

## Materials and methods

The present study was conducted in the Department of Obstetrics and Gynecology at Shri BM Patil Medical College Hospital and Research Centre, Bijapur Lingayat District Educational Association (BLDE) (Deemed to be University), Vijayapura, India.

Ethical approval was obtained from the Institutional Ethics Committee of Shri BM Patil Medical College Hospital and Research Centre (approval number: BLDE(DU)/IEC/891/2022-23), in accordance with the guidelines of the Declaration of Helsinki. The study was registered with the Clinical Trials Registry of India (CTRI/2023/10/059019).

Inclusion and exclusion criteria

Between April 2023 and February 2025, 5,221 women who gave birth at Shri BM Patil Medical College Hospital and Research Centre were assessed for eligibility to participate in the research. The study’s inclusion criteria consisted of pregnant women aged 18 years or older who had elevated blood pressure readings of ≥140/90 mmHg, measured twice six hours apart, along with significant proteinuria, defined as either ≥300 mg in a 24-hour urine analysis or ≥1+ by dipstick, occurring after 20 weeks of gestation in a previously normotensive woman. Additionally, participants were required to have a singleton pregnancy and receive prenatal care at the Bijapur Lingayat District Educational Association.

The exclusion criteria included any cases involving congenital fetal malformations or chromosomal abnormalities, chronic hypertension, twin pregnancies with PE, renal disease with PE, cardiac disease with PE, recent infections, or the presence of antiphospholipid antibody syndrome.

A total of 102 pregnant women who met the inclusion criteria were enrolled and categorized into two groups: (1) PE patients (51 cases) and (2) 51 healthy pregnant women with matched obstetric scores, age ±5 years, and period of gestation ±1 week, representing the control group.

Sample size

With an anticipated sensitivity and specificity of miRNA in PE of 90.0% and 85.0%, respectively, as reported by Tolba et al. [10], and considering the prevalence of PE at 4.6% [11], a sample size of 51 per group was required at a precision of 10% and 95% confidence level. The total sample size was 102, assuming equal-sized groups. The formula used is as follows:

 \[ N = \frac{Z^2 P(1 - P)}{\Delta^2} \]

Methods

All examined cases fulfilled the following requirements: the patient's medical information was collected to determine age, previous pregnancies, family risks, gestation period, and the onset of PE. The examination protocol included a systematic assessment of mean arterial blood pressure (MABP), blood pressure, pedal edema, weight measurements, body mass index (BMI), renal function tests (RFT), liver function tests (LFT), coagulation profile, obstetric growth scan with Doppler, and fundoscopy.

Blood Sample

Three millilitres of blood were drawn, and the serum was extracted and divided into portions before being collected. The serum aliquots were stored at −80 °C until analysis. Serum extraction required centrifugation at 1600 rpm for 15 minutes at room temperature. The supernatant from the isolation process was collected into Eppendorf tubes. A second centrifugation at 14,000 rpm for 10 minutes was performed to separate cell debris, after which the supernatants were stored at −80 °C prior to RNA extraction.

RNA Extraction

RNA was extracted from 200 µl of serum using the NucleoSpin Plasma Isolation Kit (Macherey-Nagel, Westphalia, Germany). RNA purity was assessed using a Tecan multimode plate reader (Tecan, Männedorf, Switzerland) at 260/280 OD. The RNA concentrations ranged from 0.25 to 8 μg.

Polyadenylation and Reverse Transcription

cDNA synthesis reactions were conducted for each RNA sample to enable quantitative polymerase chain reaction (qPCR) analysis. The precise quantity of miRNA levels can be quantified using a standard curve. Subsequent reagents were combined in a 0.2-milliliter tube free of RNase. The thermal cycler incubated the reactions at 37°C for one hour using its temperature programs and then shifted to 85°C for five minutes to deactivate the enzymes. Each tube was supplemented with 90 μl of ddH₂O to achieve a final volume of 100 μl. This prepared cDNA solution was used for miRNA quantification procedures (Table 1).

**Table 1:** Poly(A)/cDNA synthesis reaction RNA: ribonucleic acid, mRQ: mitochondrial ribosomal protein Q, cDNA: complementary deoxyribonucleic acid

Reagent	Volume (µl)
(2x) RNA sample (0.25–8 μg)	3.75
mRQ enzyme	1.25
mRQ buffer	5
Total volume	10

Quantification of miRNA by qPCR

The standard curve method facilitated the procedure. Two additional qPCR amplifications were performed using U6 snRNA controls for ΔΔCt analysis and cDNA from synthetic miRNA for standard curve analysis (Tables 2-3).

**Table 2:** Sample qPCR reaction qPCR: quantitative polymerase chain reaction

Reagent	Volume (µl)
TB green advantage premix (2X)	12.5
ddH2O	9
cDNA	2.0
mRQ 3’ primer (10 μM)	0.5
miRNA-specific primer (10 μM)	0.5
ROX dye (50X)	0.5
Total volume	25

**Table 3:** U6 qPCR reaction qPCR: quantitative polymerase chain reaction

Reagent	Volume (µl)
TB green advantage premix (2X)	12.5
ddH2O	9
U6 forward primer (10 μM)	0.5
ROX dye (50X)	0.5
cDNA	2.0
U6 reverse primer (10 μM)	0.5
Total volume	25

Reactions were conducted according to the protocols specified by Takara. A single-sequence qRT-PCR was performed using the real-time kit. The ABI QuantStudio 5 instrument (Applied Biosystems, Waltham, MA) was used for this procedure. The protocol included denaturation at 95°C for 10 seconds, followed by 40 cycles of qPCR: 95°C for 5 seconds and 60°C for 20 seconds. A dissociation curve was then performed with the following steps: 95°C for 60 seconds, 55°C for 30 seconds, and 95°C for 30 seconds.

Delta-Delta Ct (ΔΔCt) Method

The delta-delta Ct (ΔΔCt) method probes relative miRNA quantities in different samples by normalizing them against U6 RNA. The Ct values were calculated by analyzing the unknown miRNA of each tested sample and the reference U6 RNA. Following this measurement process, the relative levels were calculated using the ΔΔCt method.

Absolute Quantification Method (Standard Curve)

A calibrated synthetic miRNA formulation is the foundation for generating serial dilutions to establish the standard curve. The Ct values obtained during the experiments determine the miRNA copy number through the established plot. A logarithmic scale plot was employed to display Ct data from duplicate qPCR runs of cDNA derived from synthetically diluted miRNA samples and their corresponding input miRNA copy numbers (Step 1). Using the standard curve from Step 1, the corresponding RNA copy numbers were calculated by retrieving data from the average Ct values of each duplicate experiment.

Statistical Analysis

The analysis used SPSS Statistics version 20 (IBM Corp., 2011. IBM SPSS Statistics for Windows, Version 20.0. Armonk, NY: IBM Corp.). The Mann-Whitney U test was employed to analyze non-normally distributed variables, and the chi-square test assessed the statistical associations between categorical variables in the two examined groups. The Mann-Whitney U test was used to compare the expression levels of miRNA. Spearman’s correlation coefficient "r" determined relationships between miRNA-210 and other variables, ranging from -1 to +1. A p-value below 0.05 was considered statistically significant for this study. All tests performed were two-tailed. Receiver operating characteristic (ROC) analysis assessed PE through miRNA-210, determining optimal cutoff points and diagnostic metrics, including specificity, sensitivity, and positive and negative predictive values.

## Results

**Figure 1:**
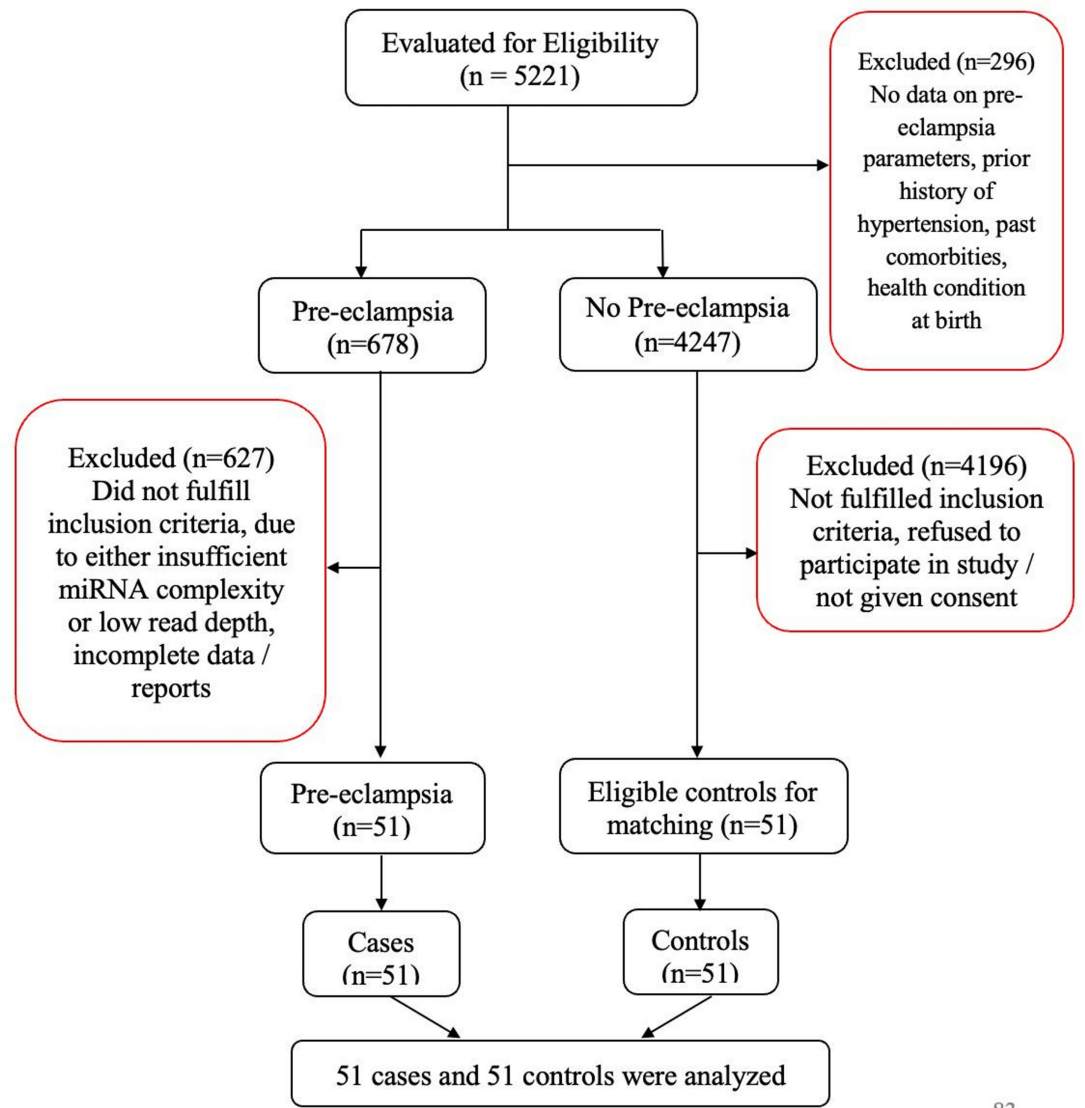
Flowchart of participant recruitment in the case–control study

From April 2023 to February 2025, Shri BM Patil Medical College Hospital and Research Centre facilitated the delivery of 5,221 women. A total of 102 consenting women who met the inclusion criteria have been enrolled in this research (Figure 1).

Table 4 displays results indicating that no substantial differences (p>0.05) existed between PE patients (cases, Group 1) and the control group (Group 2) in terms of gestational intervals, BMI, parity status, serum urea, serum creatinine, hemoglobin percentage, platelet counts, and international normalized ratio (INR) values. However, with p<0.05, systolic blood pressure (SBP), diastolic blood pressure (DBP), maternal age, serum glutamic-oxaloacetic transaminase (SGOT), serum glutamic-pyruvic transaminase (SGPT), MABP, proteinuria, alkaline phosphatase (ALP), and miRNA-210 were significantly elevated in individuals with PE compared to the control group.

**Table 4:** Comparison of clinical and lab data between the studied groups * statistically significant SBP: systolic blood pressure, DBP: diastolic blood pressure, MAP: mean arterial blood pressure, PT: prothrombin time, PTT: partial thromboplastin time, SGOT: serum glutamic-oxaloacetic transaminase, SGPT: serum glutamic-pyruvic transaminase, TSB: total serum bilirubin, ALP: alkaline phosphatase, SD: standard deviation, MG: multigravida, PG: primigravida

Variables		Cases (n=51)	Controls (n=51)	Test statistic (Mann-Whitney U)	p-value
miRNA-210 (fold change)	Mean ± SD	25.41 ± 10.13	2.05 ± 3.35	15.000	<0.001*
Maternal age (years)	Mean ± SD	24 ± 4	23 ± 3	1176.00	0.032*
Gestational age (weeks)	Mean ± SD	38 ± 1	38 ± 1	1032.00	0.089
BMI (kg/m^2^)	Mean ± SD	27.9 ± 2.2	28 ± 1.6	1244.500	0.996
Parity state	PG (n%); MG (n%)	30 (58.82); 21 (41.17%)	30 (58.82); 21 (41.17)	-	0.50
SBP (mmHg)	Mean ± SD	155 ± 15.2	114 ± 7	0.000	<0.001*
DBP (mmHg)	Mean ± SD	98.8 ± 8.6	72.7 ± 6	0.000	<0.001*
MABP (mmHg)	Mean ± SD	117 ± 9.7	86.5 ± 4.6	0.000	<0.001*
Hemoglobin (g/dl)	Mean ± SD	11.4 ± 1.7	11.3 ± 1.2	1175.000	0.743
Platelets (1000/ul)	Mean ± SD	229 ± 93	236 ± 71	1204.000	0.672
PT (sec)	Mean ± SD	10.1 ± 1.8	12.6 ± 1.2	814.500	0.44
PTT (sec)	Mean ± SD	25.3 ± 3.04	27.3 ± 2.02	709.000	<0.001*
INR	Mean ± SD	0.84 ± 0.10	0.83 ± 0.06	1280.000	0.502
Urea (mg/dl)	Mean ± SD	18.10 ± 7.2	17 ± 3.9	1299.500	0.344
Creatinine (mg/dl)	Mean ± SD	0.57 ± 0.16	0.58 ± 0.14	1219.500	0.754
SGOT (U/L)	Mean ± SD	47.1 ± 115	24.2 ± 5.06	816.500	0.041*
SGPT (U/L)	Mean ± SD	32.5 ± 97	19.2 ± 4.1	998.000	0.032*
TSB (mg/dl)	Mean ± SD	0.61± 0.41	0.43 ± 018	1020.000	0.008*
Unconjugated (mg/dl)	Mean ± SD	0.39 ± 0.35	0.25 ±0.14	1076.000	0.009*
Proteinuria (≥1+ by dipstick method)	Chi-square test	n=37 (72.54%)	0	-	<0.001*
Serum protein (g/dl)	Mean ± SD	6.27 ± 0.73	6.043 ± 0.48	1085.000	0.009*
Serum albumin (g/dl)	Mean ± SD	3.04 ± 0.45	3.57 ± 0.56	552.000	<0.001*
Serum uric acid (mg/dl)	Mean ± SD	5.6 ± 1.5	4.4 ± 0.93	670.500	<0.001*
ALP (U/L)	Mean ± SD	253 ± 92	160 ± 51.9	375.000	<0.001*

Serum miRNA-210 levels were markedly elevated in pregnant women with PE who subsequently developed the condition (mean ± SD: 25.41 ± 10.13), with a median of 23.66, compared to those without PE (mean ± SD: 2.053 ± 3.35), with a median of 0.65 (p<0.001) (Table 5).

**Table 5:** miRNA-210 levels in the study groups SD: standard deviation, PE: pre-eclampsia * statistically significant

Study groups						
With PE (cases n=51)			Without PE (controls n=51)			
Item	Mean	SD	Mean	SD	Test statistic (Mann-Whitney U)	p-value
miRNA-210 (fold) (2^- ΔΔCt)	25.41	10.13	2.05	3.35	15.000	<0.001*

**Figure 2:**
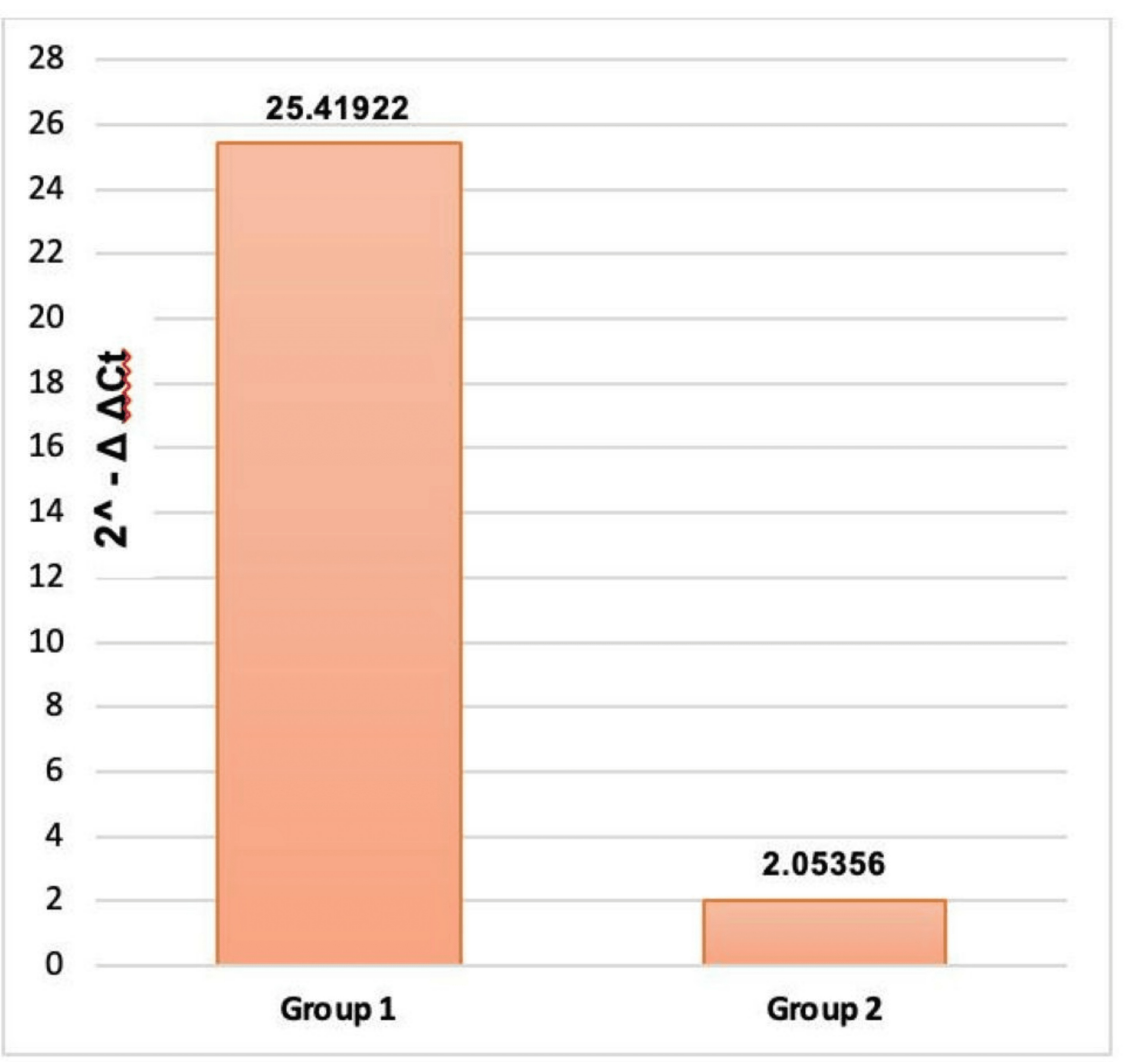
Bar graph showing miRNA-210 in the study groups

Analysis of the bar graph indicates that Group 1 patients show a 25.41-fold increase in miRNA levels compared to Group 2 patients, who exhibit a 2.05-fold increase (Figure 2).

**Figure 3:**
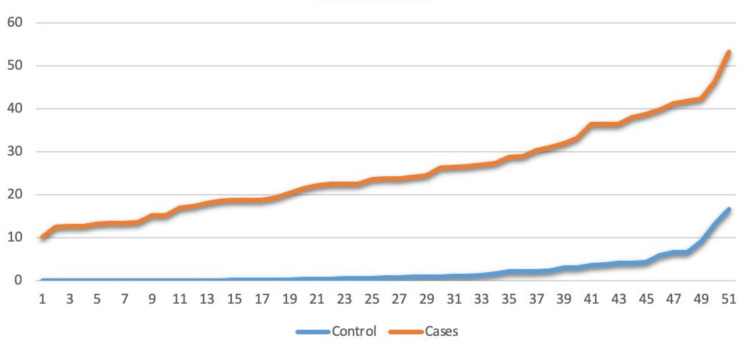
Comparison of cases and controls using miRNA-210

The results demonstrate that miRNA-210 exhibits higher expression levels in PE patients compared to normotensive healthy women (Figure 3).

Table 6 reveals a statistically significant positive correlation between miRNA-210 and gestational age, SBP and DBP, SGOT, MABP, prothrombin time (PT), SGPT, serum urea, creatinine, albumin, protein, uric acid, and ALP. ROC analysis evaluated the diagnostic efficacy of miRNA-210 in identifying cases of PE.

**Table 6:** Correlation coefficient (r) between miRNA-210 and study groups Spearman’s correlation was used. BMI: body mass index, SBP: systolic blood pressure, DBP: diastolic blood pressure, MABP: mean arterial blood pressure, PT: prothrombin time, PTT: partial thromboplastin time, INR: international normalized ratio, SGOT: serum glutamic-oxaloacetic transaminase, SGPT: serum glutamic-pyruvic transaminase, ALP: alkaline phosphatase

miRNA-210	r (correlation coefficient)	p-value
Maternal age (years)	0.701	0.487
Gestational age (weeks)	0.382	<0.001
BMI (kg/m^2^)	-0.0036	0.720
SBP (mmHg)	0.723	<0.001
DBP (mmHg)	0.739	<0.001
MABP (mmHg)	0.757	<0.001
Hemoglobin (g/dl)	0.110	0.272
Platelets (1000/ul)	-0.128	0.198
PT (sec)	0.512	<0.001
PTT (sec)	-0.190	0.055
INR	0.181	0.069
Urea (mg/dl)	0.570	<0.001
Creatinine (mg/dl)	0.014	0.893
SGOT (U/L)	0.620	<0.001
SGPT (U/L)	0.650	<0.001
Serum protein (g/dl)	0.674	<0.001
Serum albumin (g/dl)	0.328	<0.001
Serum uric acid (mg/dl)	0.532	<0.001
Serum ALP (U/L)	0.460	<0.001

**Figure 4:**
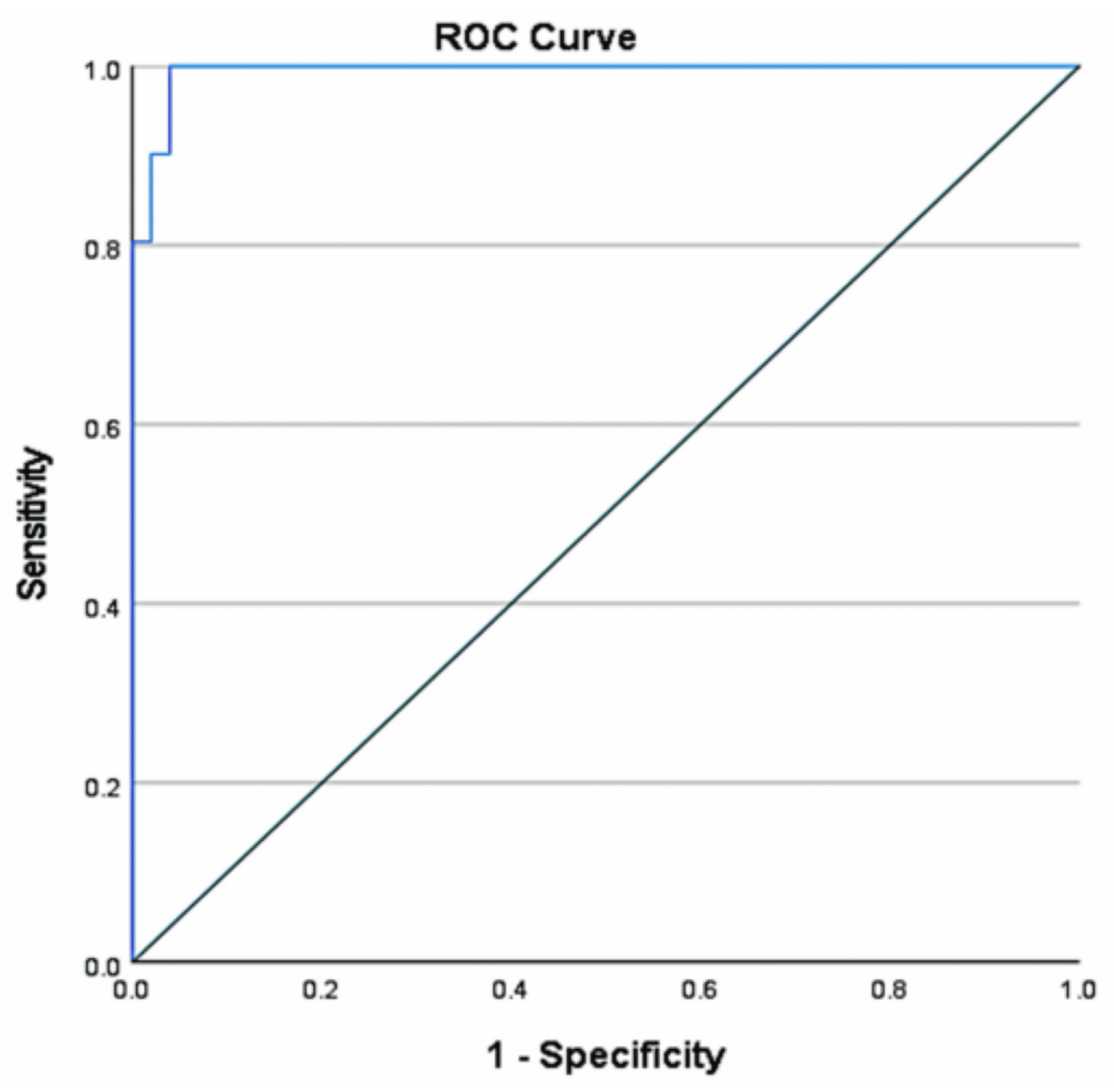
ROC curve of miRNA-210 in the evaluation of PE ROC: receiver operating characteristic, PE: pre-eclampsia

The analysis revealed a notable AUC value of 0.994, indicating a 95% confidence interval between 0.985 and 1.0. At a cutoff of 9.63 fold change, serum miRNA 210 exhibited 100% sensitivity and 96.1% specificity, with positive and negative predictive values of 96.2% and 100%, respectively, and a p-value of <0.001 (Figure 4).

Fetal growth indicators, including head circumference (311.22 ± 36.87 mm vs. 324.92 ± 9.63 mm), abdominal circumference (318.20 ± 18.66 mm vs. 327.41 ± 11.05 mm), femur length (71.25 ± 3.67 mm vs. 72.59 ± 2.99 mm), and birth weight (2.57 ± 0.49 kg vs. 2.79 ± 0.34 kg), with a p-value of <0.05, were significantly better in Group 2, suggesting improved fetal development. Meanwhile, ultrasound Doppler findings (middle cerebral artery, umbilical artery) showed no significant difference (Table 7).

**Table 7:** Fetal growth parameters in both study groups * statistically significant at p<0.05

Variables		Cases	Controls	Test statistic (Mann-Whitney U)	p-value
Abdominal circumference (mm)	Mean ± SD	318 ± 18.66	327 ±11.04	909.500	0.003*
Femur length (mm)	Mean ± SD	71.25± 3.67	72.59 ± 2.99	952.000	0.047*
Head circumference (mm)	Mean ± SD	311 ± 36.87	324 ± 9.62	814.500	0.012*
Middle cerebral artery	RI (mean ± SD)	0.76 ± 0.13	0.75 ± 0.60	1287.000	0.691
	PI (mean ± SD)	1.33 ± 0.26	1.45 ± 0.22	912.000	0.017*
Umbilical artery	RI (mean ± SD)	0.65 ± 0.10	0.63 ± 0.06	1241.000	0.446
	PI (mean ± SD)	0.96 ± 0.28	0.94 ± 0.10	1097.000	0.666
Birth weight (kg)	Mean ± SD	2.57 ± 0.49	2.79 ± 0.33	915.000	0.010*

These findings highlight that Group 1 exhibits more hypertensive and metabolic complications, whereas Group 2 demonstrates better maternal and fetal outcomes, reinforcing the benefits of the intervention (Table 4, Table 6, and Table 7).

In our study, 40 patients (39.22%) experienced maternal complications in both study groups. None of the patients had any major anesthetic or surgical complications. Out of 102 patients, all 102 had live birth newborns. Thirty-one babies required admission to the neonatal intensive care unit (NICU) for further medical management. All 102 babies were in good condition at discharge (Table 8).

**Table 8:** Maternal complications HELLP: hemolysis, elevated liver enzymes, and low platelet count, HTN: hypertension

Maternal complications	Group 1 (cases) n %	Group 2 (controls) n%
Imminent eclampsia	8 (15.68%)	0
HELLP syndrome	4 (7.84%)	0
Antenatal eclampsia	4 (7.84%)	0
Premature rupture of membranes	4 (7.84%)	1 (1.97%)
Hypothyroidism	3 (5.88%)	2 (3.92%)
Abruptio placenta	2 (3.92%)	0
Severe oligohydramnios	2 (3.92%)	2 (3.92%)
Rh-negative pregnancy	2 (3.92%)	0
Threatened preterm	0	2 (3.92%)
Anhydramnios	0	2 (3.92%)
Grade 3 HTN retinopathy	1 (1.97%)	0
HbsAg positive	1 (1.97%)	0
Total	31/51 (60.78%)	9/51 (17.65%)

We observed that a caesarean section was performed for 42 out of 51 (82.35%) births in Group 1 to prevent further maternal and neonatal complications, while caesarean sections comprised 75.5% of all births.

HELLP syndrome showed the highest mean miRNA-210 levels (34.97 ± 15.81), followed by imminent eclampsia (32.02 ± 9.2), both of which were associated with significant neonatal complications, such as respiratory distress syndrome (RDS) and birth asphyxia, requiring NICU stays of five to seven days. In contrast, lower miRNA-210 levels were observed in cases of hypothyroidism (17.61 ± 4.53) and severe oligohydramnios (17.26 ± 6.83), with either no neonatal complications or milder outcomes, such as RDS with shorter NICU stays. Conditions such as PROM (24.40 ± 7.06), antepartum eclampsia (24.04 ± 11.97), and abruptio placenta (24.04 ± 0.47) were associated with moderate miRNA-210 levels and a range of neonatal issues, including hyperbilirubinemia, birth asphyxia, and meconium aspiration syndrome, with NICU stays ranging from three to seven days. Rh-negative pregnancies had lower miRNA-210 expression (20.56 ± 2.61) and were not associated with neonatal complications (Table 9).

**Table 9:** miRNA-210 in Group 1 in association with maternal, neonatal complications, and duration of NICU stay SD: standard deviation, NICU: neonatal intensive care unit, HELLP: hemolysis, elevated liver enzymes, and low platelet count, RDS: respiratory distress syndrome

Maternal complications	miRNA-210 (mean ± SD)	Neonatal complications	Duration of NICU stay
Imminent eclampsia (n=8)	32.02 ± 9.2	Birth asphyxia (n=5)	5 days (2 neonates had prolonged stay up to 12 days)
		Meconium aspiration syndrome (n=3)	4 days
Antepartum eclampsia (n=4)	24.04 ± 11.97	Birth asphyxia (n=2)	6 days
		Meconium aspiration syndrome (n=2)	3 days
HELLP syndrome (n=4)	34.97 ± 15.81	RDS (n=4)	7 days
Premature rupture of membranes (n=4)	24.40 ± 7.06	Hyperbilirubinemia (n=4)	7 days
Abruptio placenta (n=2)	24.04 ± 0.47	RDS (n=2)	5 days
Severe oligohydramnios (n=2)	17.26 ± 6.83	RDS (n=2)	4 days
Hypothyroidism (n=3)	17.61 ± 4.53	No complications	No duration of stay
Rh-negative pregnancy (n=3)	20.56 ± 2.61	No complications	No duration of stay

Out of the 51 cases studied in Group 1, maternal complications occurred in 31 cases (60.78%) of patients, and NICU admission was necessary for 24 neonates (47.06%).

The observed variations in hematological, biochemical, and Doppler indices suggest that Group 1 may be associated with hypertensive complications and suboptimal fetal outcomes. At the same time, Group 2 demonstrates improved maternal and fetal health parameters. Our results show that increased miRNA-210 expression is directly linked to disease progression in both the mother and neonate. These findings support the role of miRNA-210 as a reliable marker for predicting PE.

## Discussion

miRNA-210 is one of several miRNAs exhibiting higher expression levels in early to mid-gestation PE placenta and maternal plasma samples. Investigating the molecular pathways of miRNA-210 activity may enhance our understanding of PE pathophysiology by uncovering potential therapeutic targets.

Our study aims to evaluate the potential of miRNA-210 as a prognostic biomarker for PE by comparing its expression levels between PE cases and normotensive controls and by correlating these levels with clinical, biochemical, and fetal growth parameters.

Our study demonstrated significantly elevated plasma miRNA-210 levels in pregnant women with PE compared to normotensive controls. This supports the growing evidence that miRNA-210 plays a pivotal role in multiple pathophysiological pathways, including cancer, oxidative stress, and apoptosis [12]. The increased expression of miRNA-210 in PE may be attributed to decreased oxygen tension at the feto-maternal interface, resulting from abnormal remodeling of maternal blood vessels by trophoblasts [13].

Mayor-Lynn et al. emphasized the functional importance of miRNA-210, showing that it regulates over 100 genes in patients with PE, suggesting its broad regulatory potential in disease pathology [14]. Our findings are consistent with the study by Jairajpuri et al., who also reported significantly higher levels of plasma miRNA-210 in PE women compared to normotensive controls [15].

The majority of the study population (59.8%) belonged to the age group of 20-24 years; 24 patients (23.52%) were in the 24-28 years age group, while 12.74% were in the 28-32 years age group, and four (3.92%) were in the 31-36 years age group. The mean age of our study population in Group 1 was 24.55 ± 4.42 years, and in Group 2, it was 23.75 ± 3.70 years. As observed in our study, a younger cohort may display different biomolecular profiles compared to older cohorts. However, our study did not directly correlate age (p=0.701, r=0.487) with miRNA-210 levels. The age profile in our cohort is consistent with other studies conducted in similar geographical regions; Mammdoh et al. also reported a predominantly young study population when assessing plasma miRNA-210 levels among at-risk women [16].

A study by Gunel et al. observed that plasma miRNA-210 levels were significantly higher in PE pregnancies than in normotensive pregnancies during gestational weeks 24 to 40 [17]. Adel et al. reported elevated miRNA-210 levels in placental and serum samples of PE primigravidas compared with normotensive primigravidas. This finding closely correlates with our study population, where a majority (58.83%) were primigravidas [7].

Ghafari et al. conducted a study involving 90 pregnant women between 26 and 40 weeks of gestation, dividing them into PE (n=48) and normotensive (n=42) groups. They found elevated plasma levels of miRNA-210, miRNA-155, and miRNA-494 in the PE group, which supports the upregulation of these miRNAs in PE [18].

Among 102 patients, antenatal complications occurred in 39.22%. However, no maternal mortality was observed in our study. A total of 17.65% of Group 2 (nine cases) had complications, while Group 1 had a higher rate of 60.78% (31 cases). The presence of complications like HELLP syndrome, abruptio placenta, and Grade 3 hypertension retinopathy exclusively in Group 1 reinforces the notion that specific pathological processes, such as endothelial dysfunction, coagulopathy, and hepatic stress, are more prevalent in patients with elevated miRNA-210 expression. This aligns with the conclusions of Tolba et al., who reported that severe PE cases exhibited a higher incidence of multi-organ involvement [10].

Fetal growth restriction occurred primarily in Group 1, with a prevalence of nine cases (19.65%). Statistical analysis produced a p-value of 0.041 (using the chi-square test), suggesting a potential connection between groups. These results indicate pathological processes such as placental insufficiency and maternal vascular vasoconstriction in Group 1 patients. Sirenden et al. observed fetal complications at a rate of 41.7% in the severe PE group with a statistical significance of p≤0.05 [19].

These findings conclude that higher miRNA-210 expression in women with PE is responsible for maternal and neonatal complications.

Association between miRNA-210 and clinical and biochemical parameters

The field of biomarker research now utilizes circulating miRNAs as promising diagnostic indicators because these molecules persist at stable levels in circulating blood.

Our findings revealed significantly elevated plasma miRNA‑210 levels in pregnant women with PE compared to normotensive pregnant women, consistent with studies conducted by Jairajpuri et al. [15], Tolba et al. [10], and Mammdoh et al. [16].

The mean miRNA‑210 level in our study's Group 1 was (mean ± SD: 25.41 ± 10.13), while in Group 2, it was (mean ± SD: 2.053 ± 3.35). This significant difference, with a p-value of <0.001, indicates a strong association between elevated miRNA‑210 and the presence of PE. The clear separation in mean values supports the hypothesis that miRNA‑210 is a critical biomarker for distinguishing between PE and normotensive pregnancies. These findings are reinforced by previous studies, including that of Jairajpuri et al. [15], who reported significant fold changes in miRNA‑210 expression between mild and severe PE (fold changes of 10.43 and 19.20, respectively, compared to controls). Similarly, Mammdoh et al. observed markedly elevated plasma miRNA‑210 levels in women with PE (mean ± SE: 19.23 ± 6.95) compared to those without (4.29 ± 1.36; p 0.001) [16].

The significant difference in mean values in our study highlights the biological importance of miRNA‑210. It suggests its potential as an early prognostic marker to identify women at risk long before clinical manifestations appear.

In both study groups, we found no statistically significant differences in gestational BMI, age, hemoglobin percentage, platelet count, PTT, PT, INR, serum creatinine, and serum urea (p-values: 0.322, 0.089, 0.996, 0.743, 0.672, 0.44, 0.502, 0.344, and 0.754, respectively) [20,21], indicating that these parameters are not confounding variables in our analysis.

According to standard diagnostic criteria, proteinuria remains a hallmark for diagnosing PE. Our study also found a statistically significant relationship (p<0.001) between proteinuria, serum uric acid levels, and PE, supporting earlier findings that associate these biochemical markers with disease severity and maternal-perinatal outcomes [22].

Additionally, our findings align with existing literature reporting elevated serum bilirubin and liver enzyme levels in PE patients, reflecting hepatic hypoxia and hepatocyte necrosis [23]. Such changes, occurring as early as the first 20 weeks of gestation, have been shown to predict severe PE and a higher risk of maternal and fetal complications [24].

Correlation analysis showed a significant positive correlation between miRNA‑210 levels and gestational age, systolic and diastolic blood pressures, MABP, PT, serum urea, SGOT, SGPT, serum protein, albumin, uric acid, and ALP (p<0.001). Conversely, we observed a non-significant negative correlation between miRNA‑210 and BMI, platelet count, and PTT (p-values: 0.720, 0.198, and 0.55, respectively), which mirrors findings reported by Tolba et al. [10].

We also performed ROC analysis to evaluate the diagnostic performance of miRNA‑210 in PE. The results revealed an AUC of 0.994 (95% CI: 0.985-1.0), with 100% sensitivity, 96.1% specificity, and positive and negative predictive values of 96.2% and 100%, respectively. These findings highlight miRNA‑210 as a highly reliable biomarker for diagnosing PE. Gan et al. conducted ROC analysis and reported an AUC of 0.750 with a 95% confidence interval, further supporting its diagnostic utility [25]. Tolba et al. [10] reported an AUC of 0.933 (95% CI), while Mammdoh et al. reported an AUC of 0.852 (95% CI) [16].

The robust positive correlations with blood pressure and biochemical parameters suggest that miRNA‑210 plays a central role in the pathogenesis of vascular dysfunction in PE. Furthermore, the inverse relationship with fetal growth parameters supports the idea that elevated miRNA‑210 may impede trophoblast invasion and placental development, leading to growth restriction. These comprehensive correlation analyses provide strong evidence for the utility of miRNA‑210 as a multifaceted prognostic biomarker.

Strengths of the study

This study stands out for its comprehensive and multifaceted approach in evaluating the prognostic potential of miRNA‑210 as a biomarker for PE. One of its primary strengths is the detailed comparative analysis between two well-defined intervention groups, which delineates clinical, biochemical, and fetal parameters. The study’s robust design incorporates molecular and clinical assessments, ensuring that the observed differences in miRNA‑210 expression are evaluated alongside critical factors such as blood pressure indices, biochemical markers, and fetal growth measurements.

The use of rigorous statistical methods, including non-parametric tests and chi-square analyses, strengthens the reliability of the findings. In addition, correlation analyses to examine the relationships between miRNA‑210 levels and various clinical parameters provide deeper insight into the underlying pathophysiological mechanisms of PE.

This integrative approach reinforces the potential role of miRNA‑210 as a prognostic tool and offers a holistic view of how maternal and fetal outcomes are interlinked. The study’s methodological rigor, comprehensive data collection, and multifactorial analysis are significant strengths, positioning it as a valuable contribution to understanding PE and developing early diagnostic strategies.

Limitations of the study

With only 102 participants, the study sample may not accurately reflect the range of demographic traits and genetic backgrounds in the broader population. Although the study focused solely on miRNA-210, other miRNAs and genes may also be upregulated in PE and warrant further investigation. Additionally, the study was conducted at a single healthcare center, limiting the variability of clinical practices and patient demographics.

## Conclusions

This study identifies that pregnant women with PE exhibit significantly elevated serum levels of miRNA-210 compared to normotensive pregnant women. Thus, serum miRNA-210 may be a non-invasive diagnostic and prognostic biomarker for PE. The results of our study enhance the understanding of the fundamental molecular mechanisms underlying PE and support future research aimed at refining diagnostic criteria and exploring targeted therapeutic interventions to mitigate the adverse outcomes associated with this condition. However, further studies with larger sample sizes are needed to validate these findings.
